# Stress and serum cortisol levels in major depressive disorder: a cross-sectional study

**DOI:** 10.3934/Neuroscience.2020028

**Published:** 2020-11-13

**Authors:** Amanda G Bertollo, Roberta E Grolli, Marcos E Plissari, Vanessa A Gasparin, João Quevedo, Gislaine Z Réus, Margarete D Bagatini, Zuleide M Ignácio

**Affiliations:** 1 Laboratory of Physiology Pharmacology and Psychopathology, Graduate Program in Biomedical Sciences, Federal University of the Southern Frontier, Chapecó, SC, Brazil; 2 Santa Catarina State University, West Higher Education Center, Chapecó, SC, Brazil; 3 Laboratory of Translational Psychiatry, Graduate Program in Health Sciences, University of Southern Santa Catarina, Criciúma, SC, Brazil; 4 Center of Excellence on Mood Disorders, Faillace Department of Psychiatry and Behavioral Sciences, McGovern Medical School, The University of Texas Health Science Center at Houston (UTHealth), Houston, TX, USA; 5 Translational Psychiatry Program, Faillace Department of Psychiatry and Behavioral Sciences, McGovern Medical School, The University of Texas Health Science Center at Houston (UTHealth), Houston, TX, USA; 6 Neuroscience Graduate Program, Graduate School of Biomedical Sciences, The University of Texas Health Science Center at Houston (UTHealth), Houston, TX, USA; 7 Laboratory of Cell Culture, Graduate Program in Biomedical Sciences, Federal University of the Southern Frontier, Chapecó, SC, Brazil

**Keywords:** major depressive disorder, chronic stress, HPA axis, cortisol

## Abstract

Major depressive disorder (MDD) is one of the disorders that most causes disability and affects about 265 million people worldwide, according to the World Health Organization (WHO). Chronic stress is one of the most prevalent factors that trigger MDD. Among the most relevant biological mechanisms that mediate stress and MDD are changes in the hypothalamic-pituitary-adrenal (HPA) axis function. Hypercortisolism is one of the relevant mechanisms involved in response to stress and is present in many people with MDD and in animals subjected to stress in the laboratory. This study aimed to investigate the levels of stress and cortisol in individuals diagnosed with MDD from the Basic Health Unit (BHU) in a small city in the western region of Santa Catarina, Brazil. Depression scores were assessed using Beck's inventory. For the investigation of stress, an adaptation with twenty-four questions of the Checklist-90-R manual was performed. The analysis of the cortisol levels in the individuals' serum was by the chemiluminescence method. Depression and stress scores were significantly higher in individuals with MDD than in control subjects (p < 0.001). Cortisol levels were also significantly higher in individuals with MDD (p < 0.05). Besides, depression scores were positively correlated with stress scores in individuals with MDD (Pearson's “r” = 0.70). Conclusion: Individuals with MDD had higher stress levels and cortisol than control subjects. The positive correlation between the levels of stress and depression in MDD individuals suggests that these conditions are related to a dysregulation of the HPA axis function.

## 1. Introduction

According to the World Health Organization (WHO), major depressive disorder (MDD) in 2004 ranked first in ranking the leading causes of non-fatal disability and third place in the global burden of disability. The estimate is that MDD will occupy the first place in 2030, among the diseases with the highest global burden [1]. In addition to being a significant cause of disability, MDD affects more than 264 million people worldwide and is the leading cause of suicides [2].

Some psychosocial factors are involved in triggering depression. Stressful events in childhood, adolescence, or adulthood are considered villains due to their ability to trigger psychiatric disorders, such as depression or depressive-like behaviors in animals [3],[4]. The manifestations are variable both by the intensity and amount of stressful episodes and the particularity of each person and how they react to stressful events. Multiple child abuse during childhood, such as sexual and physical abuse, neglect, and violence in the family, are correlated with increased vulnerability to MDD in adult life [5]–[7]. In adulthood, personal losses, marital disagreements, or other chronic stressors can become triggers for MDD development [7],[8].

MDD is a complex disorder and presents a heterogeneity of pathophysiological mechanisms [9],[10]. Chronic stress throughout life involves several biological changes, which are underlying MDD in many patients and in laboratory animals [4],[6],[9]. Stressful events interacting with genetic mechanisms become strong allies in triggering anatomical and functional changes in MDD and other psychiatric disorders [11]. Among neurobiological mechanisms associated to stress and MDD are changes in dopaminergic, serotonergic, and noradrenergic neurotransmission systems [10],[12].

Among the most relevant biological phenomena involved in chronic stress throughout life and, consequently, in MDD is the change in the hypothalamic-pituitary-adrenal axis (HPA) function [13],[14]. Hypercortisolism is a relevant feature associated with melancholic-type depression [9].

Some research points to socioeconomic conditions as important denominators influencing the onset of psychiatric disorders [15]. Although MDD is present in individuals of various social situations, it seems to increase considerably in poor populations and low socioeconomic status, suggesting that this condition is a significant risk factor for the disorder [7],[16],[17].

In addition to socio-cultural factors, some research has indicated differences in the proportion of patients and severity of MDD between urban and rural communities, suggesting that some rural populations are highly prone to MDD [18]. Šidlauskaitė-Stripeikienė et al. [19] observed a rural community high rate of depression among adults, correlated with a low level of education, low social support, and social network, and high levels of stress. Although this study's protocol did not select a specific condition for this research in MDD patients, some characteristics of the population evaluated are the predominance of individuals in a rural community and low socioeconomic status. These conditions are substantial factors underlying chronic stress and vulnerability for MDD development.

Therefore, this study aimed to investigate stress and cortisol levels in a sample of individuals clinically diagnosed with MDD in the Basic Health Unit (BHU) of a small municipality in the western region of Santa Catarina, Brazil.

## 2. Materials and method

### 2.1. Population and sampling

This is a cross-sectional study. After approval of the project by the local Ethics Committee (Protocol 183/12), 17 individuals with MDD treated at BHU and 17 control subjects, over 18 years of age, free of any pathology and without using medication for at least 30 days before the study, were selected. Both MDD and control subjects were invited to participate in the study voluntarily. All individuals received information about the research and its objectives, with free and informed consent, as specified in resolution 196/96 of the National Health Council, and freely participated in the study through consent term. Subjects with MDD, established through clinical and laboratory criteria, free of other pathologies that could compromise the research data were included in the research. Individuals who participated as controls in the study were also registered with the municipality's BHU but did not diagnose MDD. It is a small municipality, and in the period of the research, it had a population of around five thousand inhabitants [20]. The health unit organization in the city has all inhabitants registered to monitor the population's health. Subjects outside the established age range or who had other associated pathologies were excluded.

Individuals who agreed to participate in the survey were evaluated in the morning, at an appropriate location indicated by BHU. At the time of the interview and application of the questionnaires, blood was collected for the serum cortisol levels analyses.

### 2.2. Stress and depression scores

To stress levels of the participants, this study used an inventory of stress symptoms. The inventory consisted of an adaptation, with items obtained from the Checklist-90-R [21]. The Checklist-90-R Symptom Inventory (SCL-90-R) is composed of 90 (ninety) items, of which 24 (twenty-four) were elected, with a score ranging from zero to five points in each item. Items that express stress symptoms were chosen, comparing them with stress items of the Lipp and Guevara (1994) Stress Symptoms Inventory [22]. The items identified are related to symptoms of anxiety, depression, hostilities, and somatization but are not attributed only to a psychiatric disorder. The SCL-90-R was chosen because it is practical, easy to apply, and used for psychological assessment in individuals with several pathologies and evaluated at the general clinic [23].

Beck's depression inventory [24] was utilized to investigate and confirm the clinical diagnoses of depressive disorders. The inventory constituted of 21 questions about feelings present in the last 30 days. Each item presented four possibilities (between 0 and 3 points) to answer. The research considered the sum of each response's value for the total score of the assessed population.

### 2.3. Cortisol levels

For analysis of serum cortisol levels was adopted the chemiluminescence method. The immunoenzymatic assay was performed using automatic chemiluminescence equipment, model Immulite 2000, described by [25],[26]. According to the manufacturer's recommendations, adjustments were made in four replicates when starting the routine chemiluminescence analysis on this device.

### 2.4. Statistical analysis

Inventory data and plasma cortisol levels were analyzed using the parametric t-test for independent groups. A parametric test was also adopted to investigate a possible correlation between stress and depression scores, as evidenced in MDD individuals. For the correlation test, Pearson's correlation coefficient “r” was observed. The level of significance admitted for all tests was p < 0.05. The data were analyzed using the software Statistica.

## 3. Results

Some sociodemographic data of the MDD individuals and controls, such as gender, age, and education level, are shown in table 1.

**Figure 1.**
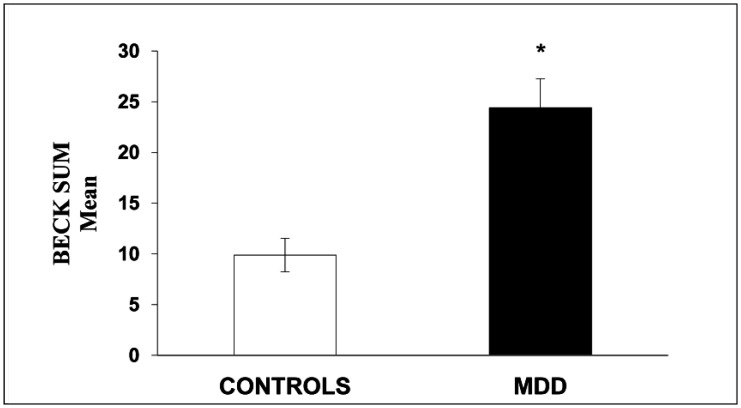
Beck Depression Inventory Scores. The results express the mean and the standard error of the mean (M ± SEM). * = p < 0.001.

Individuals clinically diagnosed with MDD at the HBS showed a significantly higher value in the sum of Beck's depression scores than the control subjects (Means ± EPM: Patients = 24.42 ± 2.86; Controls = 9.88 ± 1.65; p < 0.001) (Figure 1).

**Table 1.** Sociodemographic data of the controls and MDD individuals. F = Female; M = Male.

Group	Average years age	Gender	School Education Level				
			Incomplete Elementary	Complete Elementary	Incomplete high	Complete high	University Education
Control	48	F = 17 M = 0	8	2	0	4	3
MDD	53	F = 15 M = 2	6	6	0	3	2

**Figure 2.**
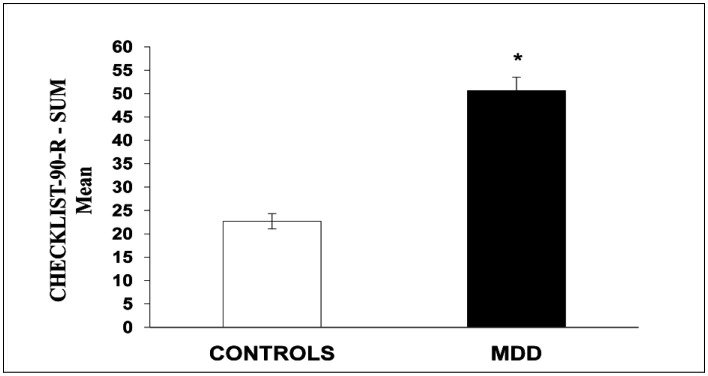
Checklist-90-R Stress Inventory Scores. The results express the mean and the standard error of the mean (M ± SEM). *p < 0.001.

The analyses also evidenced a significant difference in the sum of the scores of the stress inventory, Checklist-90-R (Means ± SEM: Patients = 50.64 ± 5.37; Controls = 22.71 ± 3.70; p < 0.001) (Figure 2).

**Figure 3.**
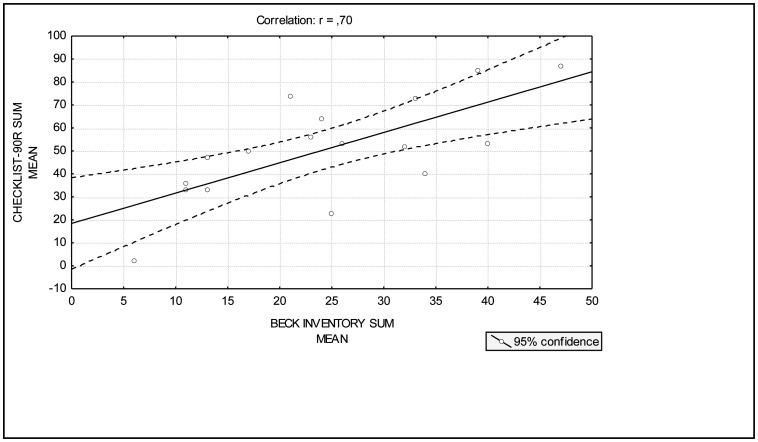
Pearson's “r” correlation between the inventories ranking sum. The results express a significant positive linear correlation. r = 0.70.

The statistical analysis also showed a significant correlation between the scores of the depression and the stress in individuals diagnosed with MDD in the clinic, with an index r = 0.70 (Figure 3). Although cortisol levels are significantly higher in MDD individuals, correlation analysis showed no significant correlation between depression scores and cortisol levels. There was also no significant correlation between stress scores and cortisol levels.

**Figure 4.**
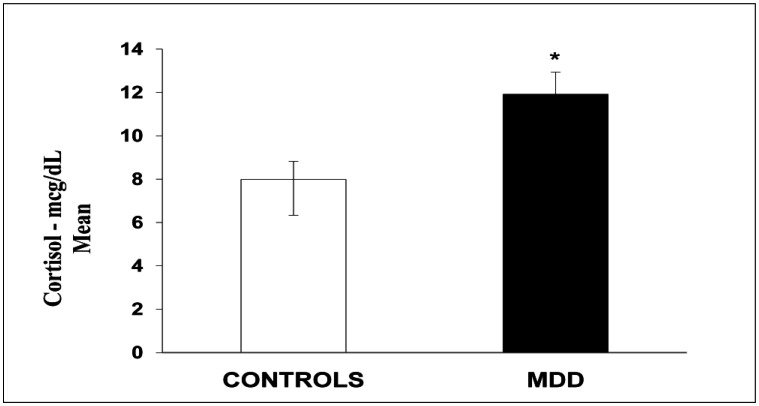
Cortisol Serum Levels. The values express the mean and the standard error of the mean (M ± SEM). *P < 0.05.

Regarding serum cortisol, MDD individuals had significantly higher levels than control subjects. Results are expressed as Means ± SEM of micrograms per deciliter of serum (mcg/dL) (Patients = 11.92 ± 1.02; Controls = 7.99 ± 0.83; p < 0.05). (Figure 4).

## 4. Discussion

This study showed a significant difference between MDD individuals and control subjects through Beck's depression inventory scores, indicating that MDD had significantly higher depression scores than control subjects. These results reinforce the clinical diagnosis of MDD obtained by individuals at the HBU.

Regarding the stress levels measured through the items listed in the Checklist-90-R inventory, MDD also had significantly higher scores than the control subjects.

The Beck depression inventory is one of the most widely used scales worldwide and has high internal consistency and validity in differentiating between MDD individuals and controls [24]. In addition to numerous empirical studies that use the inventory over time, since its construction in 1961 [27], several recent studies continue to use the inventory in several research protocols. Some authors have observed that yoga practice resulted in lower depression scores through the Beck inventory [28]. Other studies have observed that the probiotic supplement can be a useful tool in controlling mild or moderate depressive disorder and emphasized the effectiveness of using the Beck inventory as an essential tool that can be used frequently in the clinic to assist in the diagnosis of MDD [29].

Similarly, Checklist-90-R, in addition to its relevance in stress measures, is used as a tool to assess depressive symptoms, as it covers stress measures closely related to MDD. A survey of anxious and depressed patients used the Checklist-90-R to evaluate cognitive-behavioral therapy's effect on these disorders. The results suggested a beneficial effect of the therapy, corroborating the scale's usefulness in assessing depressive and anxious symptoms [30].

Depression has a profound negative effect on people's lives and health and is related to high levels of stress generated by uncertainties and low resilience levels [31]. Chronic stress is a highly relevant factor in triggering and shares critical physiological changes in MDD. Exposure to life stressors is one of the most relevant precipitating factors in developing depressive episodes [32]. It is already well defined in the scientific literature that recurrent depression can be a consequence of exposure to chronic stress events during life [6]. In this study, in addition to a significant increase in stress scores, the results of MDD individuals showed a significantly positive correlation with depression levels, reinforcing the involvement of chronic stress in disorder.

Among the most critical physiological mechanisms affected by chronic stress is the function of the HPA axis [9]. Chronic stress has a relevant involvement in the deregulation of the axis action and the recurrence and severity of MDD [33].

Although changes in the HPA axis may differ according to the MDD subtype, scientific literature has shown that chronic stress can deregulate the axis feedback system, increase cortisol levels [34], and culminate in a situation of resistance to glucocorticoids [13].

It is crucial to highlight the results in a recent meta-analysis, which found that cortisol was the only biological marker that increased the odds for both the onset and relapse/recurrence of MDD [35].

Among other functions of glucocorticoids, there is the inhibition of the transcription of inflammatory molecules, thus controlling the exacerbation of inflammatory processes [36]–[38]. However, in situations of resistance to glucocorticoids, the pro-inflammatory response becomes excessive [39]. Chronic inflammation is involved in the vulnerability of the development of MDD and other psychiatric disorders. Glucocorticoid resistance is also a factor that potentiates the severity of MDD [37],[40],[41].

Stress, both in early life and adulthood, is highly related to inflammation, mainly if caused by a feeling of possible risk and physical or social threat, commonly reported by individuals with MDD [37],[42]. Many studies also provide evidence that inflammation, both peripheral and central, is strongly associated with MDD [43].

It is also important to consider studies with evidence that individuals with low socioeconomic status submitted to stress conditions have a peripheral increase in interleukin 6 (IL-6), a pro-inflammatory cytokine [44].

Thus, the evidence in this study suggests that stress is involved with hyperactivity of the HPA axis, with increased levels of cortisol, which is possibly a relevant mechanism mediating stress and MDD in these individuals.

## 5. Limitations

A probable limitation of this study is related to the small number of individuals evaluated. However, depression scores through the inventory are clinically confirmed. Besides, the levels of stress and cortisol are indicators that strengthen the results of this research.

## 6. Conclusion

Individuals clinically diagnosed with MDD had higher scores for depression and stress-related symptoms. MDD individuals also had significantly higher levels of cortisol than control subjects. Also, depression scores were positively correlated with stress scores. These results suggest that chronic stress is involved in elevated cortisol levels, which, at least in part, appears to be the underlying mechanism of MDD in these individuals.
